# Filling New Canals

**Published:** 1884-05

**Authors:** 


### FILLING NEW CANALS.

Many substances have been used for this purpose, and dentists who, by long practice, have perfected the manipulation of any one of them, and have met with a reasonable success in their use, are too often inclined to be opinionated and dogmatical in their teachings, forgetting that their success may be due to their exceptional skill in a method which may not be in strict accordance with physiological law. The young men of the profession desire to know the best and readiest way, and not that which by long experimentation and delicate manipulation may be made to answer.

There are certain definite ends to be gained in the filling of roots of teeth, the first of which is the proper sealing of the foramen, and the thorough occupation of every portion of the vacant canal. It has been urged that if the apex be completely stopped, it matters little whether or not the rest of the root cavity be filled. To our apprehension this is an error. If vacant places be left in the canal, there may be an infiltration into them of matter which, undergoing putrefaction, may give rise to dentinal disturbances that shall hazard the preservation of the tooth. If a canal with an open foramen be not filled at all, we know that the periodical filling up of the vacant space and the subsequent degeneration of the contents, may cause a fresh outbreak of an abscess that nature had once entirely healed. This infiltration will depend in a degree upon the loose, or comparatively dense structure of the tooth itself. The filling of an ordinary root is prophylactic, and not remedial. The cavity once cleaned and made aseptic, and the sinus or abscess thoroughly evacuated, the disease is in all ordinary cases cured. But that the territory may remain in a healthy condition it is necessary that a fresh cause for an outbreak be not admitted, and the proper filling of the root is intended to secure this result. Nerve canals should then be filled with a material that can be made to conform to all the sinuosities and inequalities, the ramifications and bulbous chambers of the once living pulp.

But a filling material may fulfill all these requirements and yet be lacking in other essential qualities. It should be of an indestructible material, or it may itself, by its decomposition, contribute toward the very result which we would avoid. It need not be of an antiseptic character, because if the root and contiguous tissue be in a healthy condition antiseptics will only prove irritative. If there be no septic matter that requires to be neutralized, what necessity is there for an antiseptic? If the root be not aseptic, it should not be filled.

Every artificial root filling is a foreign substance that the tissues must be forced to tolerate. That which is of the blandest character and the least irritative will be the most likely to prove successful in doubtful cases. That which is the most foreign in its characteristics to the tissues which it is to inhabit, will be the least apt to be tolerated by teeth that are inclined to inflammatory action, and it is this very class of problematical cases that should be most steadily kept in mind. The teeth or the organizations that will accommodate themselves to almost any foreign intrusions, need not give us anxiety.

A root filling should consist of some simple substance, or if it be a compound it should be a very stable one. It is better that it be amorphous, unorganized, non-crystallizable.

If a substance made up of divers materials, that under provocation may lose their affinities for each other, be introduced into a root canal, there is danger that decomposition may take place, and in the course of the long years during which it is expected to exist under circumstances quite unfavorable to its integrity, it may fail, and the tooth be lost.

It should be of such a character and consistency that, without undue irritation, it may seal up the open ends of the dentinal tubules, and so prevent degeneration of the dentinal structure, discoloration, and putrefaction of the living matter of the tooth.

What material most nearly answers all these demands? Not a metallic one, for that is objectionable on every one of the grounds advanced, unless it be that of imperishability. Gold is practically indestructible, but is objectionable in every other way. If a combination of metals, or an amalgam be employed, it has not even the advantage of being a simple substance.

All metals are conductors of heat, and therefore liable to cause disturbances through thermal changes. They are difficult of manipulation; they are essentially foreign and unaccommodating to tooth tissues; they cannot be made to follow up tortuosities of crooked canals, or be inserted in flattened fissures, and they will not seal up the ends of the dental tubuli. In our search for the ideal filling, they must therefore be excluded.

The oxy-chlorides answer some of the conditions very well, but the usual excess of the chloride is an irritant, and therefore undesirable. It is an antiseptic, and that may recommend it to the slovenly operator (we do not mean that all dentists who use it are slovens), but we have already shown that antiseptics are undesirable. Besides, it is difficult of manipulation, because in many cases it will set and clog the canal before it is possible to get the most minute ramifications filled. There is also a possibility of its final decomposition.

The oxy-phosphates are liable to the same objection, except that they are less irritable.

Some form of the natural hydro-carbon gums instinctively suggests itself in the consideration of the subject from our present standpoint. Resinous gums have been used, but they do not answer all the ends demanded as well as those which are more inert. Of them all probably gutta-percha presents the fewest objectionable features, and to our mind comes the nearest to being the ideal filling, of anything that has been proposed. We have used it almost exclusively for some years, and with constantly increasing satisfaction. Perhaps such instances have occurred, but we certainly cannot now call to mind a case in which it became necessary to extract a tooth, through any kind of a failure of the root filling. So far as we know, its use, as it is now commonly employed, was first suggested by Dr. H. J. McKellops, of St. Louis, and to his persistent earnestness in demonstrating its excellencies the profession is deeply indebted.

The manner of its employment is very simple. Common yellow gutta-percha is dissolved in chloroform until a cream-like consistency is obtained. The Donaldson spring temper nerve broaches are the best instruments for its introduction. The nerve canal being entirely prepared for filling, the rubber dam is put in place, and with one of the broaches dipped in it, the solution is gradually pumped to the very apex of the root. As the chloroform evaporates more of the solution is worked up. The appearance of the broach will, to the experienced eye, give a very clear idea of when the end is reached. A few fibers of cotton may finally be wound about the broach, and this used as a piston to force the solution into every crevice. The patient will usually let the operator know when the foramen is reached. If a little is forced through the apex it will do no harm, further than to cause a little temporary inflammation. The absorbents will finally take care of it. We have probably caused its extrusion hundreds of times, but have yet to see the first case in which the salvation of the tooth was seriously endangered.

The cavity of decay should be temporarily stopped with cotton dipped in the solution, and the patient dismissed for a day or two, or until the chloroform shall have entirely evaporated, when the gutta-percha may be finally condensed in the mouth of the canals, and the tooth cavity filled. It is very seldom necessary to ream out canals before filling them, when this material is employed.

